# Parental Gender Affirmation Model: A culturally informed framework

**DOI:** 10.1016/j.ssmmh.2024.100304

**Published:** 2024-02-20

**Authors:** Stanley R. Vance, Luz Venegas, Jack Johnson, Anita V. Chaphekar, Anoushka Sinha, Deepika D. Parmar, Jae Sevelius

**Affiliations:** a Child and Adolescent Gender Center, Benioff Children’s Hospital, Division of Adolescent and Young Adult Medicine, Department of Pediatrics, University of California, San Francisco, 550 16th Street, San Francisco, CA, 94158, USA; b Department of Medicine, Center for AIDS Prevention Studies, University of California, San Francisco, 550 16th Street, San Francisco, CA, 94158, USA; c Benioff Children’s Hospital, Division of Adolescent and Young Adult Medicine, Department of Pediatrics, University of California, San Francisco, 550 16th Street, San Francisco, CA, 94158, USA; d Division of Adolescent Medicine, The Permanente Medical Group, Kaiser Permanente Northern California, 1425 South Main Street, Walnut Creek, CA, 94526, USA; e Department of Psychiatry, Columbia University. 1051 Riverside Drive, New York, NY, 10032, USA

**Keywords:** Gender expansive, Transgender, Non-binary, Black and Latine transgender youth, Parents, Gender affirmation, Stigma

## Abstract

Benefits of parental gender-affirming behaviors on the mental health and well-being of the broader gender-expansive youth population have been extensively documented. However, the nature and impact of these behaviors have not been explored by centering Black and Latine transgender/non-binary youth (BLTY). This article offers a new framework called the “Parental Gender Affirmation Model.” This framework conceptualizes parental gender-affirming behaviors toward BLTY through the lenses of intersectional stigma and cultural gender norms and uses the Theory of Planned Behavior and Modified Gender Affirmation Model as foundational frameworks. We analyzed qualitative data from 43 interviews with BLTY, parents of BLTY, and Black and Latine transgender/non-binary young adults from California in the United States to develop the framework. The “Parental Gender Affirmation Model” starts with behavioral antecedents and ends with impacts of these behaviors on BLTY’s well-being. This framework will inform the development of critically needed, culturally-informed interventions to support parental gender affirmation of BLTY.

## 1. Introduction

Black and Latine transgender/non-binary youth (BLTY) have multiple minoritized identities—their gender identity differs from societal expectations attributed to their sex assigned at birth, *and* they belong to minoritized racial and/or ethnic groups. They often encounter intersectional stigma, which is the convergence of stigma and systemic discrimination, including transphobia and racism (Sievwright et al., 2022; Turan et al., 2019). Experiences of intersectional stigma may amplify their vulnerability to poor health outcomes (Logie et al., 2011; Singh, 2013). The adult counterparts of BLTY experience substantive psychosocial inequities with high rates of victimization and poor health outcomes (Balzer et al., 2012; Sherman et al., 2020; Wilson et al., 2018). Only recently have the youth antecedents for these adult outcomes been explored. For example, studies demonstrate that BLTY experience high rates of depressive symptoms and suicidality, when compared to peers, with race-based and gender-based harassment as risk factors (Vance et al., 2021). Emerging data demonstrate that BLTY’s mental health may be positively affected by gender affirmation—an interactive and interpersonal process by which they receive social recognition and support for their gender identity (social gender affirmation) and/or receive medical treatment that physically affirms their gender (medical gender affirmation) (Sevelius, 2013). Parental support is a form of social gender affirmation that is a protective factor for mental health among BLTY (Vance et al., 2023).

A recent study found parental acceptance was associated with lower odds of depression among BLTY and their White peers (Vance et al., 2023). In this study, parental acceptance was measured using the parental acceptance subscale of the Parental Attitudes of Gender Expansiveness Scale for Youth (Hidalgo et al., 2017). It measures youth perceptions of their parents’ gender-affirming behaviors, such as whether their parents are proud of them, allow them to be themselves, advocate for their rights, protect them against gender-based prejudice, and support their medical transition. Other studies have demonstrated that parental gender-affirming behaviors are associated with improved mental health among gender-expansive youth (Le et al., 2016; Pariseau et al., 2019; Simons et al., 2013). Most of these studies were not conducted with racially and ethnically diverse samples or with a focus on BLTY. Moreover, scales used to evaluate these behaviors did not account for intersectional stigma and cultural considerations salient to Black and Latine communities.

Cultural gender norms provide an important context for how parents engage in supportive or unsupportive behaviors towards gender-expansive youth, as culture-based customs, religion, and social institutions are commonly grounded in gender expectations (Yu et al., 2017). Studies on general gender-expansive youth populations have described how interactions with cultural and other institutions impact how parents process their child’s gender identity disclosure, seek expertise to support their child, and advocate for their child (Meadow, 2019; Toman, 2021). Cultural norms are likely important for how Black and Latine families navigate their child’s gender journey. In one study, some Black and Latine transgender adult women reported that familial religious beliefs played a role in their parents’ non-affirming behaviors, including disownment (Koken et al., 2009). Moreover, understanding parental gender-affirming behaviors toward BLTY in the context of intersectional stigma is crucial. For example, whether they support their child’s gender journey may be influenced by fears that their child could experience compounded societal stigmatization from racism and transphobia.

Even with compelling evidence demonstrating the importance of parental gender-affirming behaviors among gender-expansive youth, exploration of specific behaviors that provide or deny affirmation for BLTY and factors that impact these behaviors is needed. For BLTY, who have multiple minoritized identities, contextualizing these behaviors in gender norms and expectations specific to their cultures is key. For these youth, identifying parental behaviors that help them feel secure in themselves, view their relationship with their parent as protective, and envision a positive future for themselves are essential to their well-being. Moreover, given the current sociopolitical polarization regarding rights for gender-expansive youth to access gender-affirming medical care and engage in school (Tanne, 2023; Waselewski et al., 2023), the parent-child relationship as a safe space is even more critical.

**Fig. 1.**
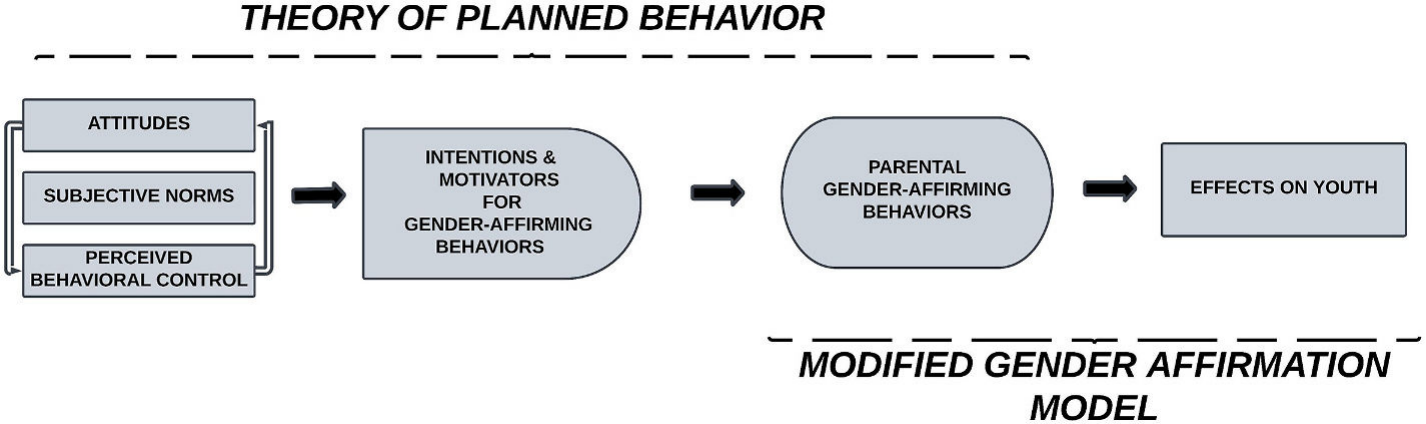
Theory of Planned Behavior and Modified Gender Affirmation Model. These foundational models are adapted and further articulated to develop the Parental Gender Affirmation Model. (1.5-column image).

The Theory of Planned Behavior (TPB) is a helpful model to guide the operationalization of parental gender-affirming behaviors towards BLTY. The TPB posits that beliefs and attitudes about a behavior predict a person’s intent and motivations to engage in the behavior (Ajzen, 1991). Fig. 1 shows the TPB with parental gender-affirming behaviors as target behaviors. Upstream antecedents influencing intentions and motivators for behaviors include attitudes, subjective norms, and perceived behavioral control. *Attitudes* pertain to the degree to which someone has a favorable or unfavorable evaluation of potential outcomes of the behavior. *Subjective norms* are perceptions about others’ approval or disapproval of a behavior. *Perceived behavioral control* involves the person’s perceived ease/difficulty in executing a behavior. These three components are precursors to *intentions and motivations* to perform a behavior.

To augment the TPB’s relevance to gender-affirming behaviors and psychosocial outcomes for BLTY, we combined it with the Modified Gender Affirmation Model. The original Model of Gender Affirmation posits that decreased access to gender affirmation coupled with an increased need for gender affirmation leads to poor health outcomes (Sevelius, 2013). Its application has focused primarily on HIV-related outcomes among transgender adults (Sevelius, 2013). With the Modified Gender Affirmation Model (Fig. 1), we conceptualize how parental gender affirmation influences BLTY outcomes.

In this paper, we utilize data from two qualitative studies to adapt and further articulate the TPB-Modified Gender Affirmation Model to create a culturally-informed conceptual framework that describes how parents of BLTY engage in gender-affirming behaviors. The goal of this paper is to articulate a framework to inform interventions to support parents of BLTY to engage in parental gender-affirming behaviors.

## 2. Methods

Data reported here were collected in two separate but similar qualitative interview studies. For the first study (Youth-Parent Study), BLTY and a related parent were interviewed separately on their perspectives regarding the youth’s gender affirmation and gender journey. For the second study (Young Adult Study), Black and Latine transgender/non-binary young adults (BLTYA) were interviewed on their childhood experiences with gender affirmation and their parents’ role in their gender journey. Therefore, data for this analysis were derived from perspectives of BLTY, parents of BLTY, and BLTYAs.

### 2.1. Participants and recruitment

The Youth-Parent Study was conducted March 2022–August 2023. The Young Adult Study was conducted August 2022–August 2023. Participants were recruited in California through passive and active recruitment methods by clinics and community-based organizations (CBOs) serving gender-expansive clients, targeted flyering, and social media campaigns. Prospective participants were informed that the research sought to understand experiences of Black and Latine transgender/non-binary youth and young adults.

For the Youth-Parent Study, to be eligible, youth needed to (1) be 14–18 years old; (2) have a gender identity incongruent with their sex designated at birth; (3) identify as Black and/or Hispanic/Latine; (4) have their gender identity known to all parents with whom they reside; (5) speak English and/or Spanish; and (6) reside and/or receive medical care in California. Youth exclusion criteria were psychiatric hospitalization in the last month and medical hospitalization in the last two weeks. Eligible parents were required to (1) be a parent of the eligible youth; (2) identify as Black or Hispanic/Latine; and (3) speak English and/or Spanish. To be eligible for the Young Adult Study, individuals needed to: (1) be 18–30 years old, (2) have a gender identity incongruent with their sex designated at birth (3) identify as Black and/or Hispanic/Latine, (4) speak English, and (5) reside in California. Exclusion criteria were evidence of severe cognitive impairment or active distress.

### 2.2. Procedures

Potential participants were screened by telephone to determine eligibility. For the Youth-Parent Study, youth were screened separately from parents. Informed consent, informed assent, and parental permission were obtained via Zoom with electronic signatures using Docusign. For the Youth-Parent Study, youth provided written informed assent if they were <18 years old or written consent if they were 18 years old; parents provided written informed consent for their own participation and written permission for their youth (if < 18 years old) to participate. For the Young Adult study, written informed consent was obtained. Enrolled participants verbally provided demographic information to the interviewer. Participants were queried on their gender identity and birth-assigned sex using open-ended questions: “What is your current gender identity?” and “What was your sex assigned at birth?” Participants were interviewed via Zoom by one research team member for approximately 60 min. Youth and parents were interviewed separately. Each participant was reimbursed with a $100 Amazon gift card upon interview completion. Interviews were audio-recorded via Zoom and audio-recordings were professionally transcribed in English or Spanish. Spanish transcripts were professionally translated. All files were encrypted and managed according to confidential and sensitive material protocols. Procedures for both studies were approved by the institutional review board.

### 2.3. Interview content

Both studies used semi-structured qualitative interviews to elicit unique perspectives. For BLTY, interviews captured their gender journey to date and perceived roles of parent(s) in their process. Parental interviews focused on their roles in the youth’s gender affirmation and supports they sought as parents. For BLTYAs, interviews were retrospective and queried their gender journey during childhood and roles of their parents. Interviews began with broad questions followed by open-ended prompts to promote expansive responses. Interview guides included probes to elicit specific information in the following domains: gender exploration (e.g., “Tell me about your process of figuring out your gender identity.”); gender disclosure (e.g., “Tell me about the first time your child told you about their gender and how you felt.”), social transition (e.g., “Tell me about your experience with social transitioning.”); gender-affirming medical interventions (e.g., “When your child expressed wanting to start medicines for their gender, what did you think of this as a parent?); and multiple minoritized identities (e.g., “Tell me how your race or culture has influenced your gender journey.”).

### 2.4. Analytic strategy

Transcripts were analyzed with Dedoose (Dedoose version 9.0.46, 2021). Team-based coding was conducted with six trained analysts under direct supervision of the senior author who has qualitative methodology expertise. Our analysis was conducted via thematic analysis; deductive and inductive approaches were employed. Study team members consisted of six people who identify as people of color and three people who identify as gender-expansive. A priori codes were based on the foundational conceptual model (Fig. 1). Initial coding consisted of reading transcripts and identifying excerpts that corresponded with a priori codes. Each interview was coded by two team members: a primary and secondary coder. The primary analyst coded each interview with a priori codes, then the secondary analyst read each coded interview while providing commentary regarding primary coding. Any differences in code application were resolved with iterative rounds of discussion. No major differences in interpretations occurred, indicating high level of coder agreement. Consistent with an inductive approach, when analysts identified new concepts not captured with a priori codes, emergent codes were created and applied across interviews iteratively.

Once all interviews were coded, searches were conducted to identify and explore relevant themes. For this analysis, the primary question was, “How do parents of BLTY engage in or arrive at gender-affirming behaviors with their child?” We searched relevant single codes across the dataset to compile excerpt reports. We also searched for convergence of multiple codes. Codes used for excerpt reports were related to youth-parent relationships, gender journey supports and challenges, and codes related to gender affirmation. Referencing the foundational conceptual model (Fig. 1), we used excerpt reports to construct extensive descriptions of themes based on unique patterns observed in the data. We then grouped longer descriptive themes into high-level themes that serve as domains in the resultant “Parental Gender Affirmation Model.” We selected quotes that represent themes and highlight participants’ perspectives using pseudonyms. The Navigating Gender Together Study Community Advisory Board comprised of BLTY, parents of BLTY, and service providers conducted a community check of themes and endorsed the resultant framework.

## 3. Results

### 3.1. Sociodemographic information

For the Youth-Parent Study, 10 youth-parent dyads completed 20 interviews; sociodemographics information is described in Table 1. The youth median age was 16.5 years (interquartile range of 1 year). In response to the gender identity question, all youth reported their gender identity as male, transgender male, female, or transgender female. Youth and their parents reported identical racial and ethnic identities. Interviews were conducted in Spanish with two parents; all other interviews were conducted in English. Twenty-three young adults completed interviews. The median age of participants was 22 years (interquartile range of 4.5 years). Table 2 presents sociodemographic information for the Young Adult Study participants.

### 3.2. Parental Gender Affirmation Model

**Fig. 2.**
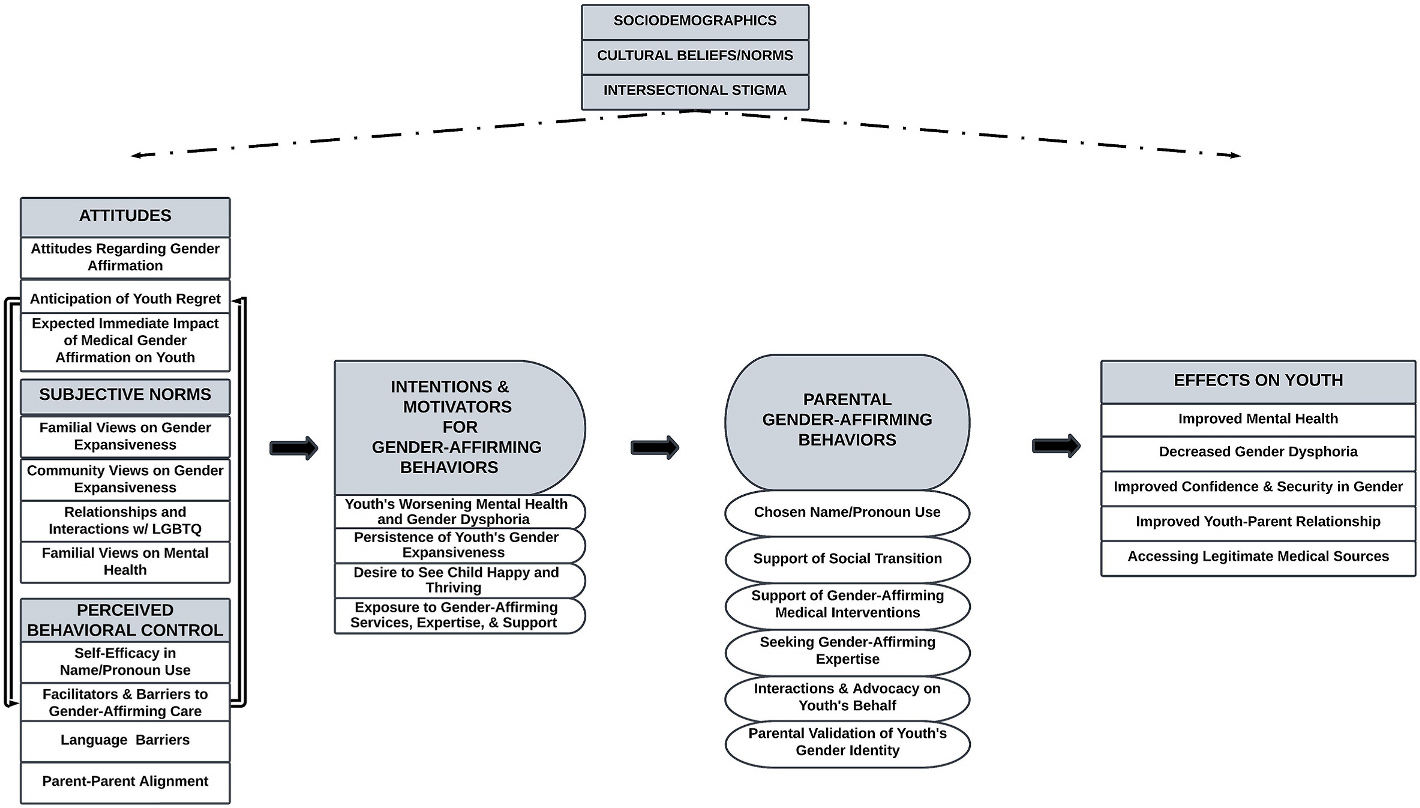
Parental Gender Affirmation Model This final resultant model further articulates foundational models using 2 qualitative interview studies. (2-column image).

Themes pertaining to behavioral antecedents, parental gender-affirming behaviors, and the impact of behaviors on youth were identified across interviews. Moreover, these themes were observed in the contexts of intersectional stigma and cultural gender norms. Our resultant “Parental Gender Affirmation Model” is displayed in Fig. 2. Here we articulate themes associated with the behavioral antecedents (attitudes, subjective norms, and perceived behavioral control), behavioral intentions, parental gender-affirming behaviors, and effects of these behaviors on youth using representative quotes.

### 3.3. Attitudes

Participants reported attitudes related to how parents evaluated and predicted the consequences of engaging or not engaging in gender-affirming behaviors. These attitudes were not static and evolved during their child’s ongoing gender journey. Multiple attitude-related themes were identified across interviews.

#### 3.3.1. Attitudes regarding gender affirmation

Parent participants reported apprehensions about their child socially transitioning and/or undergoing gender-affirming medical treatment. When youth wanted to socially transition or start gender-affirming medical interventions before age 18, they needed parental support and permission. A common source of parental apprehension towards gender-affirming medical interventions pertained to perceived irreversibility and negative physical effects.

“I was talking about how I really needed to get on testosterone. It was pretty bad by this point ’cause my dad was really hesitant to let me go on testosterone ‘cause he didn’t think as … a 17-year old, I should be doing things that could be irreversible.” John-Black Transgender Male Young Adult

One parent’s initial negative attitude towards her child starting testosterone stemmed from her worries about impacts of testosterone on her child’s future fertility. One logistical concern was about her child’s parental rights to his future child.

“The only real concern we have is he does want kids. We were originally going to look into freezing his eggs. He decided not to do it. But we have talked to him about[his] options … If he carries the baby, what are his rights to the baby?” Lucia-Latine Parent of Latine Male

Another source of parental reluctance for youth outwardly presenting as their affirmed gender was concern about the youth’s safety when being perceived as gender-expansive *and* a racial/ethnic minority, putting them at risk for experiencing intersectional stigma. In one instance, the youth, who had already socially transitioned, engaged in a typical adolescent risk-taking behavior of hanging out with friends unsupervised.

“She ended up going to an unsupervised party … It was an abandoned house … That conversation had to be … one, you’re Black, you can’t just be on anyone’s property thinking that you’re just safe and if someone sees you there that they’re going to give you the benefit of the doubt. Two, you’re also a Black trans, male to female, and the statistics say that you are the highest target for violence, and being raped, and being murdered.” Crystal-Black/Latine Parent of a Black/Latine Female

Crystal went on to describe heart-to-heart talks she had with her child about her child’s compounded risks. This discussion is similar to the “the talk” given in Black families in which they warn their teens about how police interactions can dangerously escalate because of racism (DiAquoi, 2018).

#### 3.3.2. Parental anticipation of youth regret

Participants acknowledged parental anticipation that the youth would regret physical changes stemming from gender-affirming medical interventions. This was sometimes coupled with a parent’s desire for the youth to wait until adulthood when they could independently consent to gender-affirming medical interventions. Some participants described parents having this desire because they would be absolved of responsibility if the youth ultimately had regrets. Underlying this fear was the notion that minors are too young to make such significant decisions. Early in one parent’s process of understanding her child’s desire to medically transition, she reported having frank discussions about not wanting to be responsible for the youth’s potential future regret.

“I’m opposed to being the person that makes the decision along with you and you’re just a kid. I didn’t want it to come back to me years down the line … and you’re pissed because I said ‘yeah’ and you changed your mind.” Debra-Black Parent of a Black Male

Another parent’s expectations that her child would regret changes from gender-affirming medical interventions led to her agreement with him that she would support his social transition and consider pubertal blockers, but not support testosterone, which she perceived to have more permanent effects.

“We had an agreement that we will do anything that’s not permanent like socially transitioning, assuming a new name. Because he’s so young … My contract with him was we will do social transition … I wouldn’t do permanent changes … I would want that to be his own decision.” Shauna-Black Parent of a Black Male

#### 3.3.3. Expected immediate impact of medical gender affirmation on youth

Participants reported that parents weighed the immediate benefits and risks of initiating gender-affirming medical interventions on the youth’s mental health and well-being.

“It was getting to the point where it was kind of affecting my school work; it was affecting my mental health … I was thinking about self-harm … At the end of the meeting [with my parents and therapists] I just kind of broke down and I think that was what convinced my dad.” John-Black Transgender Male Young Adult

As with John, parental agreement in pursuing gender-affirming medical interventions for the youth was often expedited by observing the youth’s worsening gender dysphoria and functioning.

### 3.4. Subjective norms

The next antecedent to parental gender-affirming behaviors in the “Parental Gender Affirmation Model” is subjective norms. Subjective norms involve an individual’s belief that people in their lives will approve or disapprove of particular behaviors. Across interviews, participants described views regarding gender-expansiveness that are held by people and communities in the parents’ lives. Moreover, participants described relationships between parents and other gender-expansive and sexual minority individuals as potential influences on their openness to supporting their child’s gender journey.

#### 3.4.1. Familial views towards gender expansiveness

Participants reported that other family members had pre-existing views towards individuals who were gender-expansive. Participants frequently grouped their families’ views towards gender expansiveness with their views towards individuals who are sexual minorities.

“When my father-in-law threw my little brother out just for being gay, like come on … I cannot fathom that. That is my blood. You know … My father-in-law can just write somebody off and not even look back, I don’t understand that.” Sade-Black Parent of a Black Female

Such views sometimes impacted the pace of the youth in disclosing their gender identity to parents. One young adult posited that within some Black communities, talking about racism was prioritized over the acknowledgement of LGBT individuals in such communities. This impacted his decision to come out, and he perceived his family’s views to be commonly held by Black families.

“Coming out to a Black family, it wasn’t really that good. Because when it comes to the Black community, LGBT things – like they’re not really talked about that often. The main topic is just like racism. When you mention LGBT, a lot of Black people –they try to like steer clear of that. So, I think it was more difficult coming out to a Black family than it would have been if I was White.” Eddie-Black Transgender Male Young Adult

#### 3.4.2. Community views towards gender expansiveness

Participants reported belonging to different communities defined by common interests, beliefs, geography, and affiliations (e.g.-religious, political). These communities often share views towards gender expansiveness and are important contexts in which parents considered how parental gender-affirming behaviors would be perceived. One parent, who was born and raised in Africa and immigrated to the United States, acknowledged negative views towards gender-expansive individuals and sexual minorities held in his hometown.

“In my hometown … most of the anti-gay/lesbian/LGBTQ rhetoric [is there and] in the capital- the big cities and the churches in Africa. [Major city in Africa] too is very backwards in that sense even though, in some senses, they’re open-minded.” Isaac-Black Parent of a Black Male

Although Isaac and his son live full-time in the US, his hometown is still an important community. Isaac later explained how he wants his son to be able to visit and wants to help him navigate safety given his hometown’s views.

#### 3.4.3. Relationships with gender-expansive or sexual minority individuals

Participants also highlighted the importance of relationships with individuals who are gender-expansive and/or sexual minorities. They reported how relationships with such individuals sometimes aided parental recognition and support of their child’s gender expansiveness.

“I don’t identify as LGBTQ, but I have a lot of friends who are. So, I think having that exposure already and friendships like that also helped me identify quicker that we had a trans child and how to be supportive.” Crystal-Black/Latine Parent of a Black/Latine Female

However, such relationships were not always helpful. In one instance, a young adult reported that her parents enlisted a gay uncle to give her advice after a psychiatric hospitalization during which the parents discarded wigs and make-up found in her room.

“They had me have a conversation with the gay uncle … He explained to me that when he was younger, he explored dressing feminine and wearing makeup, but then he would get assaulted and hate-crimed … He basically explained to me that that stuff’s all a phase … [and to focus] on having a good career so you could have a good, stable life.” Ivonne-Latine Transfeminine Young Adult

Ivonne’s uncle provided advice that aligned with the parents’ discouragement of her gender exploration. His rationale for such advice was to avoid victimization based on intersectional stigma.

#### 3.4.4. Familial views on mental health

Some participants reported familial apathy and stigma toward mental health and the need to address it through various supports, including gender affirmation.

“My family, besides my mom and dad—my aunts, uncles, and grandmas, are very Mexican and if you talk about transgender or anything like that or having depression, anxiety—they don’t think it exists.” Brandon-Latine Transgender Male Youth

“It is really hard coming to my parents, especially in like Black households … like emotionally, because we were really not close in that way … It was never mental health forward.” Caleb-Black Transgender Male Youth

Participants often described stigma associated with mental health among Black and Latine families. Notably, this stigma often made youth hesitant to disclose their gender to their families and created significant hindrances to being affirmed in their gender.

### 3.5. Perceived behavioral control

Another behavioral antecedent in the “Parental Gender Affirmation Model” is perceived behavioral control—the perceived ease or difficulty in enacting parental gender-affirming behaviors. Participants described factors that influenced parent’s perceived ability to engage in gender-affirming behaviors.

#### 3.5.1. Self-Efficacy in use of name/pronouns

Use of the youth’s chosen name and pronouns is a parental behavior that respondents acknowledged was often the first gender-affirming behavior parents were asked to enact. Parents’ perceived ease or difficulty in using the youth’s name and pronouns was impacted by different factors. Often, parents asked for more time to grow accustomed to the chosen name. Parents reported that calling a youth by their given name since birth made this challenging. Participants stated that their frustration with slip-ups did not deter them from continuing to try, especially when noticing the youth’s distress.

“That was very hard for me, not because I didn’t want to accept it, but just because when you’ve been calling someone by their name their whole life … One time he broke down … and was upset, crying. I explained to him, it’s so hard … and I told him I would do my best to work on that.” Coral-Latine Parent of a Latine Transgender Male

Some participants reported that parents held significant sentimental and cultural meaning behind the name given to the youth at birth and had difficulty with the term “dead name," specifically when the youth’s name at birth held familial significance.

“Our Mexican culture names are very … Mexicans have very like close ties to the names, so my mom picked my first name, which was Native American … My dad picked my middle name, which is just a Spanish version of like an English name.” Mateo-Latine Non-Binary Young Adult

Mateo described how the name given to him at birth had multiple cultural underpinnings for both of his parents. Parents giving names with sentimental meaning to their child is common across cultures. Our participants’ responses center the experiences of Black and Latine families and how this affects a key milestone in their gender journey: use of their chosen name.

#### 3.5.2. Facilitators and barriers to gender-affirming services, expertise, and support

Various factors facilitate or create barriers to parental gender-affirming behaviors. Participants overwhelmingly cited gender-affirming health services as critically important to their gender journeys. For some parents, access to such expertise was quite easy and was facilitated by insurance coverage of gender-affirming services. In such instances, having a knowledgeable primary care provider who knew to ask the youth about their gender identity and to whom to refer the family was paramount.

“[Her regular doctor] said, ‘No, there’s a doctor here … and the insurance will cover it.’ So, when I went there, we saw a social worker and she was the one who helped me and that’s where we connected with the doctor and that’s how everything started.” Maria-Latine Parent of a Latine Female

For others, some major barriers to accessing gender-affirming care were long waitlists to see the limited number of gender-affirming providers with expertise in evaluating and supporting gender-expansive youth and young adults.

“I waited seven months for the call. And so, during that it was me and my mom; it was us talking about if this was a for sure thing that I wanted.” Brandon-Latine Transgender Male Youth

These barriers were particularly salient for families who did not live in large cities and had to travel long distances to see healthcare providers with knowledge and expertise to support gender-expansive youth.

#### 3.5.3. Language barriers

Parents’ native language also affected the perceived ease or difficulty of engaging in gender-affirming behaviors, as it uniquely impacts use of the youth’s chosen name/pronoun and obtaining key information to support the youth’s medical transition.

“I think that my dad, who is the one who’s Hispanic, he’s Mexican … I feel like he’s probably left out a little bit because he does speak English and he does understand it, but I don’t think he understand everything … maybe doesn’t fully understands the medical terms.” Carlos-Latine Transgender Male Young Adult

Because English was not his parent’s first language, Carlos acknowledged that his father likely struggled to understand medical nuances of gender-affirming care when he first went to a gender clinic.

#### 3.5.4. Parent-Parent alignment

For youth with two parents who shared custody, parental alignment often determined whether engaging in certain gender-affirming behaviors was possible, particularly regarding decisions to pursue medical interventions for youth younger than 18 years old. In some instances, one parent being against such interventions despite the other parent and youth being in favor would prevent medical treatment. In other instances, parents would eventually agree because of family-based therapy, ongoing pleading by the youth or observing the youth’s psychological distress.

“The doctors tried to explain to him what was going on and he would say, ‘No, she needs to wait until she’s 18’ … So, [my daughter] told him, ‘Look, dad, you always think that it’s my mom, because the mother is always the one who gets blamed, but now it’s me.’ She told him, ‘Look, dad, I need you to sign this paper for me.’” Maria-Latine Parent of a Latine Female

### 3.6. Behavioral intention

Progressing through the “Parental Gender Affirmation Model,” the next domain is *intentions* to engage in parental gender-affirming behaviors. The previously discussed antecedents of attitudes, subjective norms, and perceived behavioral control will each influence parental intent to enact behaviors of interest. Intentions indicate how hard someone is willing to try to perform a behavior. Behavioral intentions include motivating factors that influence behaviors (Ajzen, 1991). Across interviews, participants described motivating factors that influenced their intentions and ultimate gender-affirming behaviors.

#### 3.6.1. Persistence of Youth’s gender expansiveness

Participants identified persistence of the youth’s gender-expansive identity and expression over time as a catalyst to engaging in parental gender-affirming behaviors.

“We honestly thought it was a phase. We did not think it would go down this path. Not that we weren’t supportive … Because one of our older daughters went through the same phase … we wanted to make sure this was something that was going to continue.” Lucia-Latine Parent of a Latine Male

As in Lucia’s case, participants reported that parents often needed to be convinced that their child’s gender identity was not a phase or due to social influence.

#### 3.6.2. Youth’s worsening mental health and gender dysphoria

A significant motivator to parents engaging in gender-affirming behaviors is observing their child’s worsening mental health symptoms. Parents described more urgency toward their child’s gender affirmation when confronted with worsening distress.

“When we found out [his dad] wasn’t really on board with it, we told [my child] that he had to wait, maybe ‘til he was 18. But then as time was going on, it was so many things related to his body dysmorphia that was causing a lot of hospital visits … That’s how all that testosterone talk came about … I told [his dad], ‘I feel comfortable with it –once we know more information about it’ … I was thinking maybe he really would go through with suicide. We might not have him by the time he’s 18.” Coral-Latine Parent of a Latine Male

As in Coral’s case, the youth demonstrating suicidal ideation and self-harm often motivated parents to increase their support of gender-affirming medical interventions.

#### 3.6.3. Desire to see child happy and thriving

Distinct from being motivated to affirm their child due to their child’s increasing distress, some parents just wanted to see their child happy and thriving. One youth described her mom’s unwavering support for her gender journey.

“She would give me advice, but she was never against [my medical transition]. I feel like it may have been hard for her at first, just cause people talk smack a lot … She went, ‘I don’t care about them. It’s about you, and I just want you to be healthy, happy.’” Tina-Latine Transgender Female Youth

“As far as her starting hormones and her body changing, I hope that she finds a really cute boyfriend that I like. I hope he’s not an asshole. Looking forward to her dating and I’m looking forward to her falling in love for the first time and maybe getting her heart broken for the first time and calling me.” Sade-Black Parent of Black Female

Sade acknowledged her child’s medical gender affirmation in the context of her child’s ability to attain typical youth milestones.

#### 3.6.4. Exposure to gender-affirming services, expertise, and support

Once parents were exposed to gender-affirming expertise and services through medical, nursing, and mental health professionals; support groups; and sources of information perceived as credible by the parents, their motivation to engage in affirming behaviors often increased. After overcoming barriers to accessing these supports, these supports were overwhelmingly seen as helpful to the parents’ motivation to engage in affirming behaviors. A youth and a parent attested to the importance of accessing this support.

“My family didn’t really know what was going on with me, like why I liked girl things and why I wanted to be a girl. So, they took me to this therapist….Thank goodness for this therapist … When I went there, I found this wonderful doctor, and she sent me to [a clinic] to start my transition at the age of 15.” Felicia-Black Transgender Female Young Adult

“I just think that we went to see the doctor and when we talked to the therapist, based on everything she tells me, I feel like she is sure …. extremely sure of what she wants to do. So, that has helped me make the decision, to help her.” Gina-Latine Parent of Latine Male

### 3.7. Parental gender-affirming behaviors

Thus far, the antecedents to gender-affirming behaviors have been described. Within the “Parental Gender Affirmation Model,” attitudes, subjective norms, and perceived behavioral control influence a parent’s intent to carry-out affirming behaviors. As seen in quotes in the prior sections describing behavioral antecedents and intentions, participants described an array of parental gender-affirming behaviors. As shown in Fig. 2, these behaviors were chosen name/pronoun use, support of social transition, support of gender-affirming medical interventions, and seeking gender-affirming expertise. Another behavior involved advocacy on the youth’s behalf, which participants described as parents defending youth against transphobia and advocating with schools and agencies for fair and inclusive treatment of their child. The final behavior was validation of the youth’s gender identity, which entails statements or actions by parents that demonstrate that they view the youth’s gender identity as valid and real.

### 3.8. Effects of parental gender-affirming behaviors on youth

The final component of the “Parental Gender Affirmation Model” pertains to the effects of parental gender-affirming behaviors on the youth. Participants reported how parental gender-affirming behaviors had positive impacts on youth well-being. In contrast, when parents did not enact affirming behaviors or enacted behaviors that were nonaffirming, participants reported deleterious effects.

#### 3.8.1. Improved mental health and decreased gender dysphoria

Participants repeatedly described how parental affirming behaviors led to improved mood, reduced gender dysphoria, and in some instances, thwarted suicidality and self-harm. One parent described the impact of parental and familial support on her son’s mental health.

“Being able to look into the mirror and feel good about what he sees in the reflection knowing that his parents and his family, his brother, his sisters love him and support him. That was life changing for him … Like it gave him a whole different kind of energy. It gave him life. I think it saved his life.” Debra-Black Parent of a Black Male

#### 3.8.2. Increased confidence and security in gender

Participants also reported that youth grew more confident and secure in their gender identity when parents engaged in gender-affirming behaviors. One parent recalled supporting her child’s gradual social transition, and her narrative culminated in her child going to homecoming as her authentic self.

“She would dress like a boy to go to school. But, when she came home, then she would take off the just plain clothes … I would buy her skirts and buy her nice clothes that she wanted. But it was mostly at home. Then, she started feeling a little bit more confident. And she would start doing videos out in the backyard with full makeup. Then, I started taking her to get manicures and pedicures … She got a lot more confident. Just recently, she went to homecoming in a gown and high heels and stockings and full makeup … I was stunned how beautiful she was.” Sade-Black Parent of Black Female

One youth reported negative effects of his parent initially not affirming his gender identity when he came out to her, which led him to feel less secure in his gender identity until later in life.

“When I mentioned that I wanted to be a boy, [my mom] laughed at me. ‘Why would you want to do that?’ And then I was like, ‘You know what? You’re right. It is kind of silly.’ And then I pushed down that part of myself until I was … this isn’t working out.” James-Black Transgender Male Youth

#### 3.8.3. Improved youth-parent relationship

Parents engaging in gender-affirming behaviors often solidified the youth-parent relationship. This was particularly apparent when the parent was proactive in seeking information related to the youth’s social and medical transition as a demonstration of their support. One youth expressed deep gratitude for her mother advocating for her and even ending relationships when her mother’s friends made discriminatory remarks.

“[My mom’s family] were all in the church most times. A few of them were like, ‘Being gay is wrong’ … But my mother has never been that way and she never will be that way. Once I came out to her and they started being like, ‘I can’t believe you would support a kid who’s in the LGBTQ community,’ Mom cut them off … I’m so grateful to have a mom like her.” Amber-Black Transgender Female Youth

Another youth reported how her father’s resistance to consenting to estrogen and not affirming her gender identity damaged their relationship.

“I don’t really count my dad’s side as my family just cause they kind of disowned me, so I don’t count them as my family.. Personally, I don’t like to talk to [my Dad]. I’m kind of forced to talk to him, which is kind of annoying.” Tina-Latine Transgender Female Youth

#### 3.8.4. Accessing legitimate sources of medical interventions

In one case, a young adult, recalled her mother taking her to a clinic that provided gender-affirming care where they hoped to start her medical transition. This parental gender-affirming behavior of seeking gender-affirming expertise was contraposed with her dad’s non-affirming behavior of refusing to consent.

“The doctor was like, ‘Yeah, we’re not going to … I would love to, but with your dad’s reaction, I’m not going to prescribe you hormones’ … We went to one of those websites, and we kind of were like, ‘Well, we’ll order whatever the doctor [mentioned].’ And I think the biggest mistake that we made at the time was that we didn’t continue to see that doctor at least to kind of oversee what was going on, because we only started me on estrogen … We didn’t start on testosterone blockers, which was definitely a mistake.” Layla-Latine/White Transgender Female Young Adult

Unfortunately, her dad refusing to support gender-affirming medical interventions led to her seeking risky sources of medical treatment, which she regretted.

## 4. Discussion

To our knowledge, this is the first study that examines parental gender-affirming behaviors specifically toward BLTY. Although prior studies have explored parental gender-affirming behaviors among gender-expansive youth, our study examined behaviors, antecedents to these behaviors and their downstream effects, specifically among BLTY through lenses of cultural norms and intersectional stigma. The TPB and Modified Gender Affirmation Model provided helpful foundations for this exploration. By further articulating these models with real-life experiences of BLTY, parents of BLTY, and BLTYAs, we developed our “Parental Gender Affirmation Model” as a novel framework that can guide development of culturally-informed interventions to support parents of BLTY in affirming their children.

The “Parental Gender Affirmation Model” starts with behavioral antecedents that are fundamental to behavioral intentions and eventual behavioral change. When a youth discloses their gender identity and their parent considers whether they will support their gender journey, parents have *attitudes* regarding if this support will be beneficial or harmful. Moreover, parents consider whether important communities in their lives will be supportive of their decision to affirm their child. These *subjective norms*, often anchored in culture-based views about gender expansiveness, affect the parent’s perceived social acceptability of affirming their child. Parents also weigh how easy or difficult it will be to engage in these behaviors. This *perceived behavioral control* is influenced by factors that parents perceive empower them to perform such behaviors and barriers that make such behaviors more challenging. These antecedents influence how motivational factors influence parent’s *behavioral intent* to engage in gender-affirming behaviors. Our model then articulates the downstream effects of parental gender-affirming behaviors on BLTY well-being.

Prior studies on general gender-expansive youth populations have described ways parents have supported their child’s gender journey such as aiding their social transition, using their chosen name/pronouns, seeking gender-affirming expertise, and advocating for their child across institutions (Meadow, 2019; Toman, 2021). Our study highlighted that such affirming behaviors are also salient for BLTY. Moreover, earlier studies demonstrate associations between parental gender affirmation and improved mental health outcomes among gender-expansive youth, in general (Le et al., 2016; Pariseau et al., 2019; Simons et al., 2013). Our qualitative data corroborates emerging evidence that parental gender-affirming behaviors also positively impact BLTY well-being (Vance SR et al., 2023). Our study contextualizes these behaviors and their antecedents within cultural gender norms/expectations and intersectional stigma. For example, Black and Latine parents expressed their concern that as their child progressed on their gender journey that their child could be targeted by multiple sources of societal stigma. Parents already recognized their child’s risk for experiencing racial and/or ethnic discrimination and were concerned about their child also being at risk for transphobic victimization. Some parents’ concerns were further reinforced by their perception of subjective norms within their families, communities, and religious institutions, such that transphobia seemed pervasive in these domains. Participants also reported deeply embedded cultural meaning behind birth names and how language barriers influenced parental perceived behavioral control in supporting their child’s gender journey. Many participants in our study noted that parents ultimately were able to affirm their child’s gender. Our data demonstrates that cultural norms and intersectional stigma are deeply embedded in the complex progression towards parental gender-affirming behaviors. In the development of interventions aimed to support parents of BLTY in affirming their child, these factors must be addressed.

Our framework provides multiple behavioral antecedent targets for interventions to promote parental gender-affirming behaviors towards BLTY to improve well-being among BLTY. To change attitudes toward the behavior, subjective norms, and perceived behavioral control, parental beliefs must be addressed (Ajzen, I., 2006). Parental psychoeducation about benefits and risks of social transition and gender-affirming medical interventions for BLTY may aid in promoting favorable attitudes towards supporting their child’s gender affirmation. Despite extensive literature demonstrating benefits of gender affirmation among gender-expansive youth, in general, scant literature addresses the experiences of BLTY until recently (Vance SR et al., 2023; Vance et al., 2021). With respect to targeting subjective norms, bolstering community connectedness and peer support among parents of BLTY may provide social networks that support gender expansiveness and counter social pressures from less supportive communities. Interventions that reduce barriers to gender-affirming services may bolster parents’ belief that they can successfully affirm their BLTY and boost their perceived behavioral control.

Data for our study were collected from specific samples of Black and Latine youth, young adults, and parents. Consistent with our goal of learning how parents of BLTY arrived at and engaged in gender-affirming behaviors, our recruitment approach focused on clinic and CBOs serving gender-expansive youth as primary venues of recruitment. Our findings may not be generalizable to youth, young adults and parents who do not have access to gender-affirming clinics, CBOs, or social networks. Also, our study may have limited generalizability to individuals of other racial and ethnic backgrounds and who live outside of California. Moreover, our Youth-Parent Study allowed for fluency in either English or Spanish, but the Young Adult Study was limited to English speakers. This limitation also has implications for the generalizability of our findings. Finally, we chose to focus on the shared experiences of minoritization among Black and Latine individuals instead of differences between racial and ethnicity groups; we recognize that Black and Latine individuals have culturally heterogenous experiences.

Despite these limitations, our study presents a novel, culturally grounded framework with significant implications. The “Parental Gender Affirmation Model” provides an opportunity to expand research focused on gender-expansive youth and finally center a framework on the experiences of Black and Latine gender-expansive youth. Our framework can be used to develop culturally-informed interventions that bolster parental gender-affirming behaviors towards BLTY as one approach to supporting their well-being. Vulnerable youth populations like BLTY are disproportionally affected by the ongoing US mental health crisis. Approximately 50% of BLTY experience depressive symptoms, suicidality, and anxiety symptoms (Vance et al., 2021, 2023). Black and Latine youth look to parents to help them navigate racism, poverty, and other challenging circumstances and promote self-esteem (Bagley and Carroll, 1998; Porta et al., 2016; Zimmerman et al., 2000). As gender-expansive youth are seeing their rights to gender-affirming health care and participation in school activities being increasingly restricted (Tanne, 2023; Waselewski et al., 2023), augmenting the parent-child relationship and aiding parents in affirming their child is even more critical. The next step in our research is to develop interventions for BLTY and their parents. We will further develop and articulate the “Parental Gender Affirmation Model” during intervention development and implementation phases. For broader implications, our framework has constructs that can be further assessed quantitatively. Future research is needed to test pathways proposed in our framework by using quantitative measures of behavioral antecedents and constructs with a larger sample of BLTY and parents. It will also be critical to explore how our framework may be applied to BLTY who are in foster care or who have parental figures that are other relatives. Additionally, although our framework was developed by centering experiences of BLTY, parents of BLTY, and BLTYAs, it is important to consider how it may generalize or be adapted for families with gender-expansive youth of other racial or ethnic backgrounds.

In summary, “Parental Gender Affirmation Model” is informed by the perspectives of multiply minoritized youth and their families. Our future work will focus on further developing the model in the context of intervention development for BLTY and their families. Additionally, we hope to disseminate and adapt our framework so that it can more broadly inform research focused on improving the lives and well-being of gender-expansive youth.

## Figures and Tables

**Table 1:** Youth-parent study participant demographics.

	Youth (N = 10)	Parent (N = 10)
	n (%)	n (%)
**Gender Identity**		
Male/Transgender Male	7 (70%)	2 (20%)^b^
Female/Transgender Female	3 (30%)	8 (80%)^b^
Designated Sex at Birth		
Female	7 (70%)	8 (80%)^b^
Male	3 (30%)	2 (20%)^b^
Race and Ethnicity^a^		
Latine	6 (60%)	6 (60%)
Black	5 (50%)	5 (50%)
**Currently on Gender-Affirming**	7 (70%)	0 (0%)
**Medications**		
**Marital Status**		
Married or in Domestic Partnership	n/a	5 (50%)
Divorced/Separated	n/a	2 (20%)
Single/Never Married	n/a	3 (30%)

A total of 20 interviews were conducted with 10 youth interviews and 10 parent interviews.

a More than 1 category could be reported; total percentage is more than 100%.

b All interviewed parents were cisgender as their gender identities and birth assigned sexes were congruent. Of the parents, 8 were cisgender females and 2 were cisgender males.

**Table 2:** Young adult study participant demographics.

	N = 23
	n (%)
**Gender Identity** ^a^	
Male/Transgender Male/Transmasculine	8 (35%)
Female/Transgender Female/Transfeminine	8 (35%)
Non-Binary	7 (30%)
Gender Non-Conforming	1 (4%)
**Designated Sex at Birth**	
Female	14 (61%)
Male	9 (39%)
**Race and Ethnicity** ^a^	
Latine	14 (61%)
Black	10 (43%)
White	5 (22%)
American Indian/Alaska Native	2 (9%)
Southeast Asian	3 (13%)
**Currently on Gender-Affirming Medications**	16 (70%)

a More than 1 category could be reported; total percentage is more than 100%.

## Abbreviations

BLTY: Black and Latine transgender/non-binary youth
BLTYA: Black and Latine transgender/non-binary young adult
TBP: Theory of Planned Behavior
